# InDels Identification and Association Analysis with Spike and Awn Length in Chinese Wheat Mini-Core Collection

**DOI:** 10.3390/ijms23105587

**Published:** 2022-05-17

**Authors:** Zhenyu Wang, Zhongyin Deng, Xingchen Kong, Fang Wang, Jiantao Guan, Dada Cui, Guoliang Sun, Ruyi Liao, Mingxue Fu, Yuqing Che, Chenyang Hao, Shuaifeng Geng, Xueyong Zhang, Peng Zhou, Long Mao, Shaoshuai Liu, Aili Li

**Affiliations:** National Key Facility for Crop Gene Resources and Genetic Improvement, Institute of Crop Science, Chinese Academy of Agricultural Sciences, Beijing 100081, China; wangzhenyu89@126.com (Z.W.); 15093595930@163.com (Z.D.); kongxingchen1990@126.com (X.K.); wangfang1213@126.com (F.W.); xidagjt@126.com (J.G.); cuidada2018@163.com (D.C.); spy5920@126.com (G.S.); liaoruyilue@163.com (R.L.); fumingxue0101@126.com (M.F.); 15670535279@163.com (Y.C.); haochenyang@caas.cn (C.H.); gengshuaifeng@caas.cn (S.G.); zhangxueyong@caas.cn (X.Z.); pzhou@caas.cn (P.Z.); maolong@caas.cn (L.M.)

**Keywords:** InDel, GWAS, spike length, awn, *TaAGL6*

## Abstract

Diversity surveys of germplasm are important for gaining insight into the genomic basis for crop improvement; especially InDels, which are poorly understood in hexaploid common wheat. Here, we describe a map of 89,923 InDels from exome sequencing of 262 accessions of a Chinese wheat mini-core collection. Population structure analysis, principal component analysis and selective sweep analysis between landraces and cultivars were performed. Further genome-wide association study (GWAS) identified five QTL (Quantitative Trait Loci) that were associated with spike length, two of them, on chromosomes 2B and 6A, were detected in 10 phenotypic data sets. Assisted with RNA-seq data, we identified 14 and 21 genes, respectively that expressed in spike and rachis within the two QTL regions that can be further investigated for candidate genes discovery. Moreover, InDels were found to be associated with awn length on chromosomes 5A, 6B and 4A, which overlapped with previously reported genetic loci *B1* (*Tipped 1*), *B2* (*Tipped 2*) and *Hd* (*Hooded*). One of the genes *TaAGL6* that was previously shown to affect floral organ development was found at the *B2* locus to affect awn length development. Our study shows that trait-associated InDels may contribute to wheat improvement and may be valuable molecular markers for future wheat breeding

## 1. Introduction

Common wheat (*Triticum aestivum* L.) is a leading cereal as a staple food for more than 35% of the world’s population. China is the largest wheat producer and consumer in the world, with more than 23 million hm^2^ of planting area in 2020. Over the past half a century, the total output of common wheat in China has increased from 20 million tons to 134.25 million tons, while the total sown area reduced from about 25 million hectares to 23 million hectares (http://www.stats.gov.cn, accessed on 3 March 2022). Therefore, the increase in yield per unit area was the principal contribution to the increase in total production. The systematic breeding of wheat based on Chinese landraces and introduced foreign cultivars has helped greatly in Chinese wheat improvement [1,2,3].

From more than 23,090 wheat accessions stored in Chinese GeneBank that were collected across wheat growing regions in China, a mini-core collection of 262 Chinese wheat accessions, representing the genetic diversity of Chinese wheat, was constructed by phenotyping and SSR genotyping [4]. The collection consisted of 157 Chinese landraces (CL) and 105 cultivars, including 88 modern Chinese cultivars (MCC) and 17 introduced modern cultivars (IMC) [5]. Significant phenotypic differences were detected between CL and MCC [5]. Using SNP markers, 6.7% of the wheat genome was found to fall in selection sweeps between landraces and cultivars and genes known for yield improvement were identified using genome wide gene association study [5]. In plants, SNPs, rather than insertions/deletions (InDels), were commonly used to identify genomic variations that affect observable phenotypes.

Abundant evidence demonstrated that genetic variations caused by InDels play a large role in phenotypic variances that affect a series of important agronomic and quality traits in crops. For example, in a genome-wide association study (GWAS) with salt tolerance in 182 wild soybean accessions, a 7-bp deletion in the promoter of *GsERD15B* (early responsive to dehydration 15B) that significantly affected salt tolerance in soybean [6] was identified. GWAS of maize drought tolerance at the seedling stage also identified 83 genetic variants, involving 42 candidate genes [7]. The peak GWAS signal showed that the natural variation in *ZmVPP1*, encoding a vacuolar-type H^+^ pyrophosphatase, contributes most significantly to the trait, which is caused by a 366-bp insertion in the promoter that contains three *MYB* cis elements critical for drought tolerance [7]. In another case, a 1-kb insertion in the upstream of the barley *HvAACT1* gene coding region enhanced Aluminum tolerance by increasing its expression and altering the location of expression to the root tips [8]. Despite many studies using SNPs in wheat, the distribution and population genetic characteristics of InDel variants in wheat have not been systematically studied.

The development of sequencing and assembly technology has shifted the limits of the reference genome in wheat research and breeding [9], greatly promoting researches in wheat evolution, domestication, selection, adaptation and genetic locus underlying traits development [10,11,12,13,14]. A genome-wide InDel-based study on the molecular basis of agronomic traits is still needed, especially for identifying novel QTL (Quantitative Trait Loci) other than those classical ones. Recently, 287 wheat accessions were used to identify 76,952 InDels. These InDels caused a frame shift in 2083 genes, including Ppd-D1 and GS5-1 and 182 rice homologs that have been functionally studied, demonstrating that InDels can impact on important functional genes [5]. The frequency of these frameshift InDels in modern cultivars (0.22) was significantly higher than that in landraces (0.17), suggesting that they were under artificial selection. With the release of the latest version (v2.1) of the wheat reference genome [15], further study on the effect of InDels on the population characteristics and phenotype of wheat should provide new insights into wheat evolution and breeding.

Wheat spike architecture is one of the important agronomic traits. Unripe spikes significantly contribute to photosynthesis and are the closer source of assimilates to caryopses, contributing to grain filling and thousand grain weight [16,17,18]. Morphological variations in spike shape (square or speltoid), length, and compactness are correlated with grain size and spikelet number per spike. Modifying spike morphology can increase grain number and size, thus improving yield [19]. Spike length affects compactness and biomass. In wheat, the domestication gene *Q*, *TB1* homolog, AP2 transcription factor *WFZP*, and *TaHOX4* all affect inflorescence architecture and development [20,21,22,23]. To identify genetic loci associated with spike length, a large-scale GWAS identified 26 QTL associated with spike length [13]. Another 39 QTL related to spike length were identified among the Chinese wheat mini-core collection [5]. These QTL provide a basis for further discovery of spike determinant genes. Awn is another key spike morphological feature. Awns play important roles in seed dispersal and crop production. The awns of wheat carry the abilities of photosynthesis and carbon exchange [24]. Up to date, three loci *B1* (*Tipped 1*), *B2* (*Tipped 2*), and *Hd* (*Hooded*) were reported as dominant suppressors for awn development [24,25]. At least one of them, B1, can be attributed to InDels for their origination [26].

Here, we describe a map of InDel variation containing 89,923 InDels derived from exome sequencing of 262 Chinese wheat mini-core accessions, which we studied regarding their divergence and selection between landraces and cultivars. The effect of InDels on population structure, principal components, and selective sweeps was observed. Further GWAS identified novel genetic loci associated with spike and awn length. Our study shows that trait associated InDels may contribute to wheat improvement and may be valuable molecular markers for future wheat breeding.

## 2. Results

### 2.1. Genomic Features of Wheat InDels

**Figure 1:**
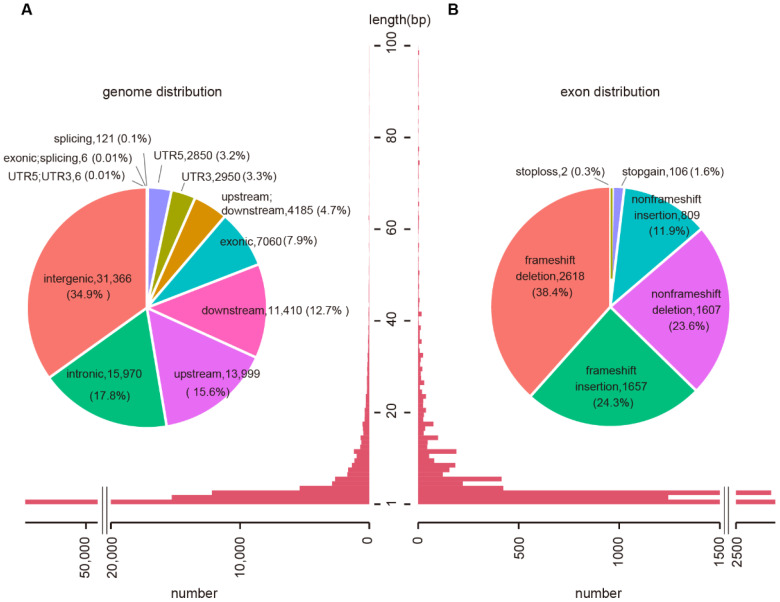
InDel annotation and size distribution. (**A**) InDel annotation (up) and length distribution in the wheat genome (down). (**B**) InDel annotation (up) and length distribution (down) in exons.

From 287 exome-sequenced wheat accessions, a total of 983,262 SNPs and 76,952 short InDels were identified with minor allele frequency (MAF) ≥ 0.05 and the missing rate ≤ 0.2% using the IWGSC wheat genome assembly RefSeq v1.0 [5,9]. Here, we re-analyzed the InDels using the newly released IWGSC wheat reference genome v2.1 [15]. A total of 89,923 InDels were identified using the Genome Analysis Toolkit (GATK) protocol with similar selection parameters. These InDels were mainly located in intergenic regions (34.9%), followed by introns (17.8%), upstream (15.6%), downstream (12.7%), and exons (7.9%; Figure 1A). The average size of InDels was 4.58 base pairs (bp), of which 96% ranged from 1 to 20 bp. Only 0.27% InDels were longer than 100 bp.

The length distribution of InDels was decreased when the length of InDels was increasing (Figure 1A). Although the length of InDels in coding regions (CDS) tend to be a multiple of 3 that may not cause frameshift, 62.7% InDels caused frameshift mutations. Among them, 38.4% were deletions and 24.3% were insertions (Figure 1B; Supplemental Table S1). The number of InDels with a base number of 3 or multiple of 3 was much higher in genic regions (23.6%) than those in intergenic regions (11.0%), indicating purified selection of InDels in genic regions.

Among 18,445 InDels located in 5 kb upstream of genes, we identified 10,039 InDels with cis-regulatory elements present nearby (≤50 bp). These InDels potentially affected 403 transcription factor binding sites, including ARF, AGL, NAC, and SPL (Supplemental Table S2), totaling 6167 genes, such as *TGW6*, *GS5-3*, *Ghd7*, *Glu-A3* (Figure S1A; Supplemental Table S3). InDels in UTRs, exons and in splicing regions often caused gene reading frame changes or amino acid loss. We analyzed these types of InDel and found that they affected 6002 genes, including important ones such as *ARF12*, *GIF1*, *Vrn1*, *GS3*, *GW7*, and *Ppd1* (Figure S1B; Supplemental Table S4). Cis-elements and gene structure associated InDels affected a total of 11,140 genes, of which 4125 were in the A subgenome, 4664 in the B subgenome, and 2351 in the D subgenome (Figure S1C).

The distribution of InDels along chromosomes was uneven (an average of 7.3 InDels per Mb with the maximum ones up to 121 InDels per Mb), with a higher density in the distal regions of chromosomes than that in the vicinity of the centromeres (Figure S2). This distribution feature was consistent with the distribution of SNP along chromosomes [14], probably due to higher recombination frequency at the outermost chromosomal regions than those near the centromeres [27]. The number of InDels in homoeologue groups was proportional to the length of chromosomes. For example, length of chromosomes in homologue groups 1 were B > A > D, the corresponding InDel numbers were also B > A > D (Figure S2), and so was InDel densities (Figure S2). Such an observation may be caused by tandem repeats and TE amplification (the main reason for a chromosome becoming longer) in these regions.

### 2.2. Population Structure of Chinese Mini-Core Collection Based on InDels

**Figure 2:**
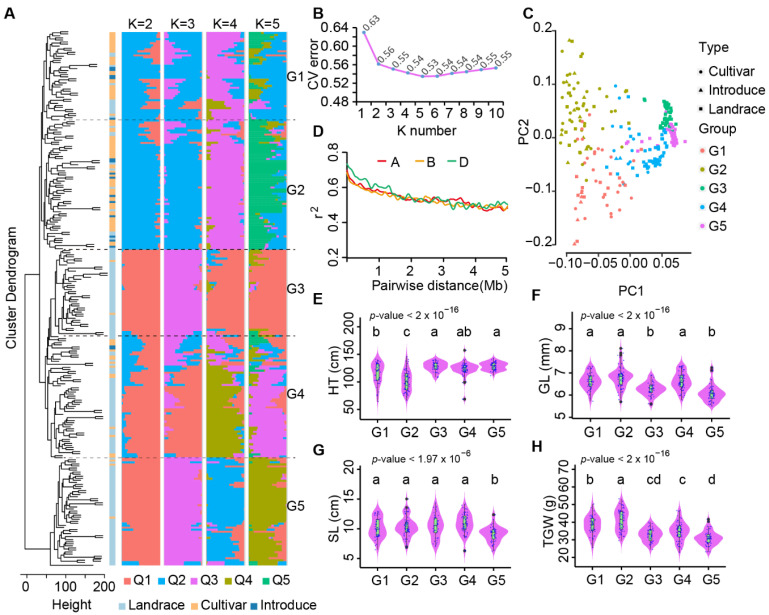
Population structure of the Chinese wheat mini-core collection using InDels. (**A**) Cluster dendrogram (left) and STRUCTURE (right) analysis separated cultivars from landraces mainly. (**B**) The cross validation (CV) error between sub-populations. The CV error between sub-populations was the lowest when the population was divided into five sub-population. (**C**) Principal components analysis (PCA) plot for all accessions. (**D**) Decay of linkage disequilibrium (LD) in the three sub-genomes. (**E**–**H**) Phenotypic differences between subpopulations. *p*-value for F statistics in ANOVA was marked in the upper left of the panel. The Tukey’s HSD test results were shown above the violin plot. There were significant differences between groups with different letters (*p*-value < 0.05).

Cluster dendrogram and population structure analysis using InDel data showed Chinese mini-core collection were divided into several subpopulations according to different clustering levels and the number of subpopulations (K) in structure analysis (Figure 2A). The cross validation (CV) error analysis showed that the CV error between subpopulations had no drastic changes when K ≥ 2, and CV error reached the minimum value (0.53) when K = 5 (Figure 2B). Following K = 5, Chinese mini-core collection was classified into five subpopulations (G1–5) that was different from the previous study based on SNPs (Li et al., 2022). Among the five groups, G1 and G2 consisted of 64.5% modern cultivars, 14.0% introduced modern cultivars and 21.5% landraces, while G3, G4 and G5 consisted of significantly more landraces, up to 86.5%, with percentages of cultivars at 12.3% for modern cultivars and 1.29% for introduced cultivars (Supplemental Table S5). The coordinated presence of G1–5 in PCA was consistent with the results from the cluster dendrogram and population structure analysis (Figure 2C). In addition, a slow LD decay curve line was observed, comparable to that derived from SNP markers [13,14] (Figure 2D).

We then analyzed phenotype variance and found significant differences among subpopulations (Figure 2E–H). For plant height, the G2 group, which included well-known accessions such as the Yangmai158, Zhengmai9023, Yannong15 and Xiaoyan6 varieties, was significantly shorter in plant height than the other four groups. The average plant height of G3, G4 and G5 were all at the same level (Figure 2E). For spike length, the G5 group, including landraces Baimangmai, Lanhuamai, and Youmangbaifu, was significantly shorter than those of the other groups. Thus, InDels are useful markers for analyzing wheat population structures to present the characteristics and phenotypic distinctions.

### 2.3. Estimation of Molecular Diversity of Chinese Mini-Core Collection Accession Using InDel Markers

**Figure 3:**
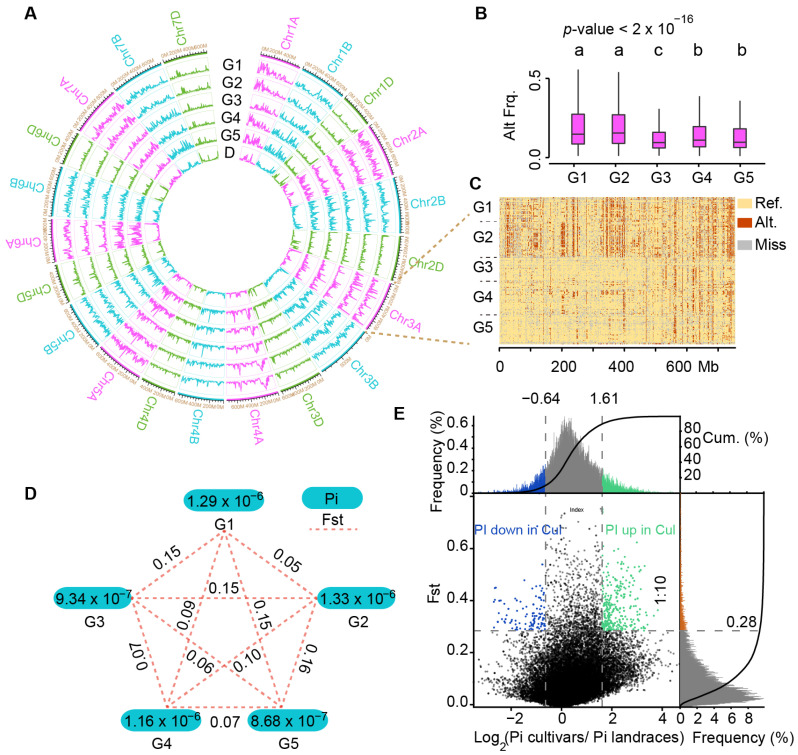
Molecular diversity of the Chinese mini-core collection. (**A**) Circle graph display of alternate allele frequency (G1–G5) and InDel density (D, density) in each sub-group. (**B**) Difference of alternate allele frequency among subgroups. *p*-value for F statistics in ANOVA was marked in the upper left. The Tukey’s HSD test results were depicted above the violin plot. There were significant differences between groups with different letters (*p*-value < 0.05). (**C**) Allele frequency of Chromosome 3A. (**D**) Dissection the molecular diversity of Chinese mini-core collection by subgroups. (**E**) Genomic diversity signatures. The top 10% of Fst and the two-tail top 10% of Pi were used as thresholds to identify distinct diversity segments between cultivars and landraces.

Alternate allele frequency analysis showed significant differences between subgroups (*p* < 2 × 10^−16^). The alternate allele frequencies of G1 (0.20) and G2 (0.20) were higher than those of G3 (0.12), G4 (0.15) and G5 (0.15) (Figure 3A,B). Chromosome 3A was then used as an example to clearly illustrate this distinction. As shown in Figure 3C, G1 and G2 showed a visibly higher alternate allele (0.19 and 0.20) frequency than G3 (0.09), 4 (0.11), and 5 (0.11) in a long chromosome region (about 1–550 Mb) in Chr3A that included several important functionally known genes in wheat, such as *TaMFT-A1* for seed germination [28], *TaGI1* for photoperiodic flowering [29]), *TaFT2* for flowering time [30] and *TaGS5-A1* for thousand-kernel weight [31]. Additional homologs to functionally characterized rice genes included *Gn1 (CKX2)* for spikelet number determination [32], *SRS3* for grain length [33], and *TGW6* for grain weight and increased yield [34]. The observations indicated that these loci may be selected during breeding, which was supported by the presence of 11 selection sweeps from landraces to cultivars and nine pedigree-based haplotype blocks with cumulative length, 60 Mb, as shown by [14].

We subsequently used InDels to calculate nucleotide diversity (π) and fixation index (Fst) among accessions of the Chinese mini-core collection. The results showed that G3 (9.34 × 10^−^^7^) and G5 (8.68 × 10^−^^7^) groups showed slightly lower levels of nucleotide diversity than others (Figure 3D). By π and Fst, G1 and G2 could be grouped together, while G3, G4 and G5 formed as a second group (Figure 3D), consistent with the results of the cluster dendrogram (Figure 2A), population structure (Figure 2A), and SNP variation frequency (Figure 3B). In fact, G1 and G2 contained 79.5% cultivars (called G1,2 group), while the remaining three groups (G3, G4, and G5, hence G3–5 group) represented 86.5% of landraces. The π ratio (π_G1,2_/π_G2–4_) and Fst (tiled every 200 kb in 1Mb window) was calculated between G1,2 group and G3–5 group, which revealed genomic diversity signatures along the 21 chromosomes (Figure 3E). A total of 284 Mb and 134.2 Mb highly divergent chromosome fragments were identified for G1,2 and G3–5, respectively. Interestingly, the cultivar-enriched G1,2 group had higher nucleotide diversity in 284 Mb selected regions that that of G3–5, while it was vice versa in the 134.2 Mb regions. In both cases, only a few percent were derived from the D subgenome while the remaining were from the A and B subgenomes (Supplemental Tables S6 and S7), indicating that A and B subgenomes are more diverse than the D subgenome, consistent with the observation from regular SNPs.

### 2.4. InDel-Based Genome-Wide Association Study (GWAS) on Wheat Spike Length

**Figure 4:**
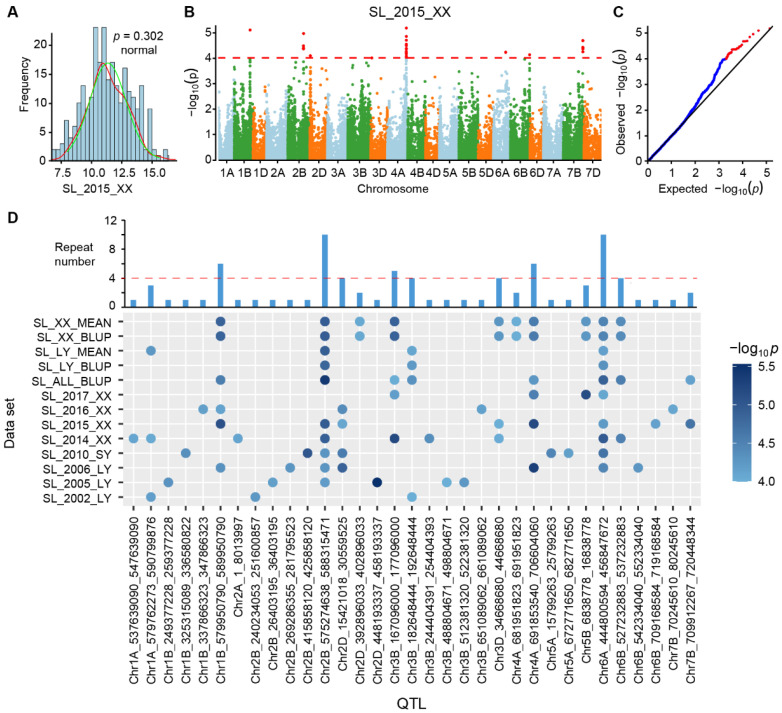
GWAS-derived QTL for spike length. (**A**) Spike length distribution and normal distribution test results. The red line corresponds to the normal distribution of spike length. The green line corresponds to the estimated best-fit normal density curve. (**B**) Manhattan plot of significant InDels by GWAS. Dashed line indicates the significance threshold (−log_10_*p* = 4). (**C**) Quantile-Quantile plots of significant SNPs. (**D**) Duplicate information for GWAS-derived QTL. The top bar graph shows the number of repetitions, and the bottom dot graph shows the details of the detected environment.

As indicated by the cluster dendrogram and population structure, which indicate that accessions in the Chinese mini-core collection can be divided into five subpopulations, we applied the first five PCs for GWAS using the mixed linear model against 13 sets of trait values for spike length traits (including the BLUP and mean values) in the Genome-wide Efficient Mixed Model Association (GEMMA) toolkit. The phenotypic data of spike length conformed to a normal distribution (Figure 4A). A total of 87 significant (*p*-value < 1.0 × 10^−^^4^) InDels were identified to be associated with spike length (Figure 4B) [35]. Quantile-quantile (QQ) plot of the data showed an acceptable separation of the observed from the expected (Figure 4C). Due to the strong linkage disequilibrium in common wheat genome, significant InDels with adjacent distances less than 5Mb were incorporated into the same GWAS-derived QTL. A total of 33 GWAS-derived QTL were identified, of which 15 overlapped with reported QTL (Figure 4D and Supplemental Table S8). Five of the QTL were replicated more than four times in different environments (Figure 4D). A total of 3236 genes were located in GWAS-derived QTL, 334 of which were known genes (Supplemental Table S9), including several for spike length, such as *OsER1* [36]. GO enrichment analysis showed that these genes were significantly enriched in GO:0010455-positive regulation of the cell fate process (*p*-value = 6.77 × 10^−^^6^), GO:0016998-cell wall macromolecule catabolic process (*p*-value = 1.32 × 10^−^^5^), and GO:0045493-xylan catabolic process (*p*-value = 1.87 × 10^−^^5^) (Figure S3), suggesting their potential effect on cell wall development.

We then scrutinized the top 2 GWAS-derived QTL (Chr2B:575274638-588315471 and Chr6A:444800594-456847672) that were detected in ten environments. On Chromosome 2B, the highest peak marker was InDel:583315471 with −log_10_*p* = 5.42, and overlapped with a reported spike length QTL, Chr2B_578399456 (Figure S4A, Supplemental Table S8). Accessions with the deletion genotype for the peak InDel:Chr2B_583315471 had a significantly shorter spike than those with the reference genotype in cultivars (Figure S4B). Moreover, InDel:Chr2B_583315471 was located in a stable LD block that spanned 4.98 Mb from 581,531,948 to 586,515,317 and contained 14 genes expressing in spike (TPM more than 1 in spike) (Figure S4C,D; Supplemental Table S9). These genes were considered as candidates contributing to the effect of the QTL Chr2B:575274638-588315471. On Chromosome 6A, the sole peak (−log_10_*p* = 4.97) at the middle was located in a LD block (from 450,071,383 to 455,065,356) (Figure S5A,B). Accessions represented by peak InDel: Chr6A_450503278 showed significant differences in spike length (*p* < 0.05) in both landraces and cultivars (Figure S5C). The LD block harboring peak InDel contained 21 genes expressed (TPM > 1) in spike (Figure S5D). These genes were considered as candidate genes contributing to the effect of the QTL Chr6A:444800594-456847672 (Figure S5D; Supplemental Table S9) that can be further verified.

### 2.5. Identification of an Awn Inhibitor at the Tipped 2 (B2) Locus by GWAS

**Figure 5:**
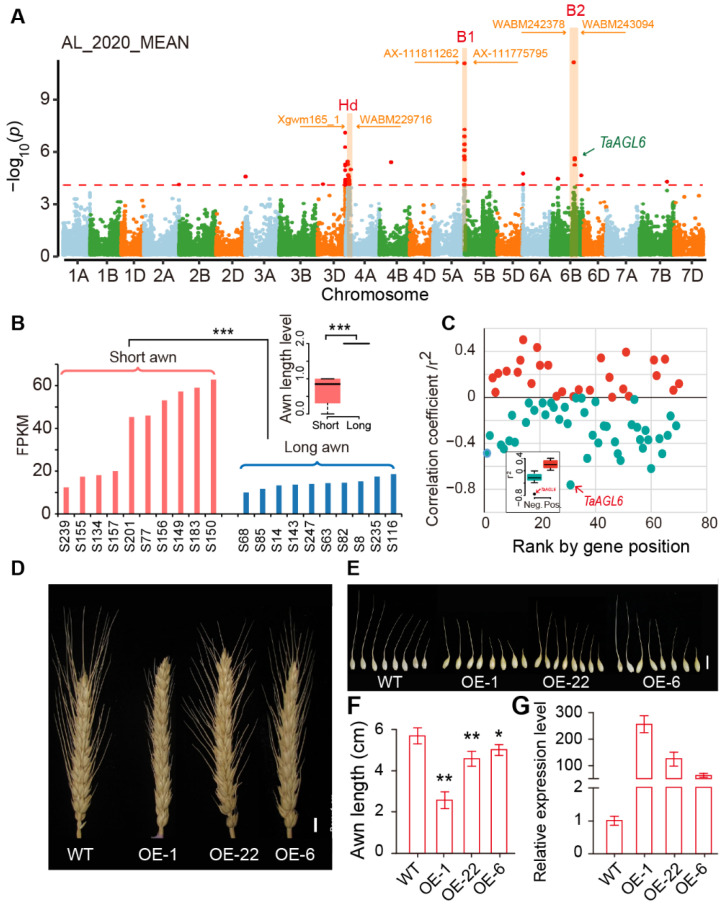
GWAS for wheat awn length and identification of *TaAGL6* as a causal gene candidate for the *B2* locus. (**A**) Whole genome Manhattan and QQ plots for loci significantly associated with awn length. Dashed line indicates the significance threshold (−log_10_*p*  = 4). The three orange shaded peaks were three awn-related loci *Hd*, *B1* and *B2*. (**B**) *TaAGL6* showed significant differential expression (*** *p* < 0.001) between 10 long-awned accessions and 10 awnletted/awnless accessions. Correlation analysis between gene expression levels and awn length in 20 accessions with inflorescence RNA-seq data as in (**C**) showing *TaAGL6* having the smallest negative coefficient (r^2^ = −0.78). (**D**–**G**) Overexpressing *TaAGL6* in cv. “Fielder” led to awnletted spikes. OE-1, OE-22, and OE-5 are *TaAGL6* overexpression lines. * *p* < 0.05, ** *p* < 0.01.

Awns are stiff, bristle-like structures extending from the tip of floret lemma in wheat and are selected during domestication and breeding due to their contribution to drought resistance and yield [37]. We investigated major peaks associated with awn length and identified three major peaks on chromosomes 5A, 6B, and 4A, respectively, which overlapped with previously reported *B1* (*Tipped 1*), *B2* (*Tipped 2*) and *Hd* (*Hooded*) QTL that were known as dominant suppressors for awn development [24,25] (Figure 5A; Supplemental Table S8). *B1* has recently been cloned as a C_2_H_2_ transcription factor with an EAR domain of transcription repression functions [26,38,39,40]. The most significant (−log_10_*p* = 12.85) InDel in the *B1* locus in our study was Chr5A_700804911, located at 19.8 kb upstream of this C2H2 gene.

We then focused on the *B2* locus on chromosome 6B for which the causal gene has not been cloned. We examined the genes which fell within 5-Mb distance of the significant InDels (−log_10_*p* = 11.06 of peak InDel). Genetically, *B2* is one of the three awn suppressors. Thus, we studied the RNA-seq data that generated from young spikes and compared with their expression patterns between two pools with 10 long-awned and 10 awnless accessions, respectively (Figure 5B). We identified TraesCS6B03G0828100, the wheat MADS-box 6 gene (*TaAGL6*), as an outlier that was most negatively correlated with awn length (r^2^ = −0.76) (Figure 5C). Importantly, *TaAGL6* was expressed at high levels in young inflorescences of awnletted accessions, while its expression was low in long-awned accessions (Figure 5B), consistent with its genetic role as a dominant repressor for awn development [25]. We then overexpressed *TaAGL6* in cv. Fielder and found that awns in transgenic plants were significantly reduced in length (Figure 5D–G), strongly suggesting *TaAGL6* as a candidate gene for the *B2* locus.

## 3. Discussion

### 3.1. InDel Diversity Was Comparable between Wheat Cultivars and Landraces

Common wheat originated in the Fertile Crescent of the Middle East. Chinese landraces are a branch in the process of world wheat dispersal and may be low in diversity relative to the accessions in the world as a whole. In contrast, modern Chinese cultivars have integrated extensive genetic germplasm from a wide range of resources, including international varieties, making them more diverse relative to landraces. Since the beginning of wheat breeding in China, introduced cultivars, such as Mentana, Funo, and Abbondana from Italy, Early Premium from US, and Lovrin 10 from Europe, as well as cultivars from CIMMYT, were widely used as founder parents [3]. Here, in our study, at the InDel level, we found that the diversity of cultivars was indeed comparable to that of landraces (Figure 2C and Figure 3D). This result is consistent with reported diversity between landraces and cultivars at the SNP level [5,14], suggesting that breeding activities were important for self-pollination crops such as wheat with increased diversity and had greatly expanded the genetic basis of modern Chinese cultivars. Similar trends of expanded genetic basis in cultivars were also observed in the international wheat collection, as reported elsewhere [41].

### 3.2. InDels Are Effective Supplements to the Analysis of Population Genetic Variation

The second-generation sequencing technologies perform large-scale sequencing, allowing the detection of large-scale mutations. Among the mutations detected, SNPs are the largest in number and most widely used in population research [42], evolutionary domestication analysis [10,43], genome-wide association analysis [44], and QTL localization [45]; however, they do not represent the whole variation of the genome. InDel, as a small fragment of variation, can cause direct damage to gene coding functions or gene regulatory regions. A large number of insertions/deletions as well as large fragment structural variants (CNV, SV, Pvals) also have a wide range of biological significance and population structure characteristics in the genome [46,47,48]. Some of them can directly affect the phenotype of crops [49,50]. However, due to the limitation of technology and cost, these variants have not been used as widely and extensively as SNPs in functional genome research. In Arabidopsis, Liu et al. (2021) developed InDelEnsembler to detect large InDels in 1047 Arabidopsis whole-genome sequencing data and discovered novel phenotypic InDels of size > 50 bp that cannot be found in previous studies [51]. In wheat, due to the extremely large size of wheat genome, it is very difficult to find large InDels in the whole genome, let alone discover InDels directly affecting the phenotypes. Here, although we did not identify InDels on the causal genes, we indeed found that the population structure and genetic diversity reflected by InDels were consistent with SNP results, indicating that InDels can also be well used in the study of wheat population structure. By comparing the correlational analysis results of InDels and SNPs, we found that 36% of the QTL in spike length and 71% of the QTL in awn length were also detected by SNPs. These well-known loci overlapped with QTL that were detectable mostly in multi-environments, demonstrating the utility of InDels for GWAS research and in novel loci determination. Moreover, Chinese wheat mini-core collection can be divided into two groups, either by InDel or SNP, representing landraces and cultivars, respectively [5]. However, with the increase in the number of population components, InDels can help dissect them into additional groups, as shown by differences in their phenotypes, demonstrating that InDels can provide extra genetic information related to phenotypes and agricultural traits. Although InDels at genic regions, such as transcription factor binding sites, may cause more severe biological effects, their presence in the genome is far lower than the SNP variations. InDels are thus supplemental to SNPs, not replacing SNPs in diversity analyses.

### 3.3. InDels Associated with Awn Length Traits

Wheat awns carry the abilities of photosynthesis and carbon exchange, which are responsible for yield in certain conditions [24,52]. At present, many QTL related to awn development have been mapped in rice, such as *An-1*, *An-2*, *An-4*, *An-6*, *An-7*, *An-8*, *An-9*, and *An-10* [53,54]. However, it is difficult to clone the genes in these QTL. Only the genes in *An-1* and *An-2* have been cloned [53,54], and some other awn-related genes have also been reported, such as *RAE2*, *OsYABBY* and *OsETT2* [55,56].

In wheat, *B1* (*Tipped 1*), *B2* (*Tipped 2*) and *Hd* (*Hooded*) have been known as genetic loci for awn development as early as 1940 [57]. However, the gene underlying *B1* has not been cloned until recently as a C_2_H_2_ transcription factor with an EAR domain of transcription repression functions [26,38,39,40]. Sequence polymorphisms in the *B1* coding region were not observed in diverse wheat germplasm, whereas a nearby polymorphism was highly predictive of awn suppression [38]. Here, the *B1* locus has been mapped by a peak InDel with a high *p*-value (−log_10_*p* = 12.85), demonstrating the validity of our InDel analysis in some way. Additional 28 significant InDels in the region may help in further mining the functional variations in the gene.

The causal genes for *B2* and *Hd* are still unknown. One of the main reasons for the difficulty in cloning wheat awn genes is the large LD interval at these regions. InDels extended the polymorphic intervals. Thus, besides SNPs, InDels further facilitate final causal gene identification. By referring to the experience of *B1* cloning and the negative correlation between gene expression levels and awn phenotypes, we identified *TaAGL6* as a candidate causal gene for the *B2* locus. Our work therefore demonstrates the significance of InDels in wheat population studies and in the application of InDels in wheat breeding.

## 4. Materials and Methods

### 4.1. Sampling and Phenotyping

Two hundred and sixty-two (262) accessions from the Chinese mini-core collection were as described previously [5]. Phenotyping of spike length (SL) was investigated in eight environments, namely 2002, 2005 and 2006, at Luoyang in Henan province, 2010 at Shunyi District, Beijing, and 2014, 2015, 2016 and 2017 at Xinxiang in Henan province. Awn length were determined using a grading standard: zero stands for no awn;1 means that the awn length is less than or equal to 4cm; 2 means that the awn grows more than 4cm. All accessions were planted in an experimental field in Beijing with an arrangement order design including three replicates.

### 4.2. Sequence Capturing and Sequencing

Total genomic DNA from seedling was extracted with a Plant DNA Mini Kit (Aidlab Biotech, Beijing, China). The exon capture array designed by Jordan et al. was used, and the probes were obtained from Roche NimbleGen (http://www.nimblegen].com/products/seqcap/ez/designs/, accessed on 1 May 2021); the exon capture procedure is the same as that published by Jordan et al. [58]. The Illumina HiSeq X-ten platform was used to generate 46.66 billion paired-end reads with 150-bp read length.

### 4.3. Sequence Quality Checking and Filtering

The original data of the next-generation sequencing carried the adapter sequence that was added when the library was built. It is necessary to remove adapter contamination and low-quality value bases (both ends of the reads) and reads containing low quality values above a threshold level before data processing. In this study, reads with the following conditions were deleted: those containing *n* greater than 10%, those with the number of bases of phred quality < 5 accounting for more than 50%, and those with a length less than 120 bp.

### 4.4. Sequence Alignment and InDel Detection

The filtered raw data were aligned to the newest reference genome RefSeq v2.1 [15] using BWA (Burrows–Wheeler Aligner, Version: 0.7.17-r1188) software, with parameters: ‘mem-t 4-k 32-M’ [59]. Samtools (Version: 1.9) was used to convert the alignment results from the SAM file format to the BAM file format [60]. The low-quality reads of the alignment results were removed: (1) the quality value was greater than 10; (2) the mismatch was less than 5; (3) the PCR redundancy was removed; (4) the multiple alignments (≥2 hits). Subsequently, InDel calling was performed with the Genome Analysis Toolkit (GATK, version v4.0) by the HaplotypeCaller method [61]. Finally, variations that passed the quality filter (recommended parameters in GATK: -filter “QD < 2.0” --filter-name “QD2” \-filter “QUAL < 30.0” --filter-name “QUAL30”\-filter “FS > 200.0” --filter-name “FS200” \-filter “ReadPosRankSum < −20.0” --filter- name “ReadPosRankSum-20”) and met a miss ratio ≤ 0.3 and MAF ≥ 0.03 in the population were further used for phasing genotypes and imputing ungenotyped markers using Beagle(Version:4.1) software [62]. Finally, InDels that met a miss ratio ≤ 0.2 and MAF ≥ 0.05 in the population were used in the remaining analyses. Variation annotation was performed using the ANNOVAR (Version: 2013-05-20) software package based on the reference genome RefSeq v2.1 gene annotation information [63]. Here, the term “upstream” and “downstream” is defined, respectively, as the 2-kb region from the starting codon ATG or 2-kb away from the stop codon. If a variant is located in both downstream and upstream regions (possibly for two different genes), then the “upstream, downstream” will be printed in the output.

### 4.5. Population Genetics Analysis

Cluster analysis among materials used Hierarchical Clustering. Population structure was calculated by the Expectation Maximization algorithm (EM) based on ADMIXTURE software [64]. The number of populations from 2 to 5 (genetic clusters K) were assumed in the calculation process, and 10,000 iterations were used for each estimation. Plink (v1.90b6.10) software was used to perform Principal Component Analysis (PCA) and Linkage disequilibrium (LD) coefficient (r^2^) calculations with parameters ‘--bfile --pca’ and ‘- -ld-window-r2 0 --ld-window 99,999 --ld-window-kb 1000′ [65]. To reduce the impact of environmental differences at different experimental sites on GWAS, we performed Best Linear Unbiased Prediction (BLUP) on the phenotypic data using R lme4 package (Version:3.2.2). A sliding-window approach (500 kb windows sliding in 200 kb steps) was applied to quantify polymorphism levels (π, pairwise nucleotide variation as a measure of variability), and genetic differentiation (Fst) among sub-groups by vcftools software [66].

### 4.6. GWAS Analysis

Only InDels with MAF ≥ 0.05 and missing rate ≤ 0.2 in the population were used in the GWAS. An association analysis was performed using the genome-wide efficient mixed-model association (GEMMA) software package [67]. The population structure was represented by the first five principal components as fixed effects. In addition to the spike length value of eight environments, BLUP and MEAN values were applied to GWAS analysis. XX_BLUP: Best linear unbiased prediction (BLUP) value of phenotypic data collected from Xinxiang in 2014, 2015, 2016, 2017. XX_MEAN: Mean value of phenotypic data collected from Xinxiang in 2014, 2015, 2016, 2017. LY_BLUP: Best linear unbiased prediction (BLUP) value of phenotypic data collected from Luoyang in 2002, 2005 and 2006. LY_MEAN: Mean value of phenotypic data collected from Luoyang in 2002, 2005 and 2006. ALL_BLUP: BLUP value of phenotypic data collected from Xinxiang (2014, 2015, 2016, 2017), Luoyang (2002, 2005, 2006) and Shunyi (2010). BLUP was used to calculate the breeding values with lme4 packages in R.

### 4.7. Construction of TaAGL6 Overexpression Transgenic Lines

The construction method of *TaAGL6* overexpression transgenic lines refers to Kong et al. Briefly, the pUbi:TaAGL6 construct was developed from the full-length ORF of TaAGL6 with a 6× myc tag at the N terminal. It was then cloned into the reconstructed binary vector pCAMBIA3300 containing the maize ubiquitin promoter [68].

### 4.8. Statistical Analysis

The R language comes with its own function, two-tailed Student’s *t*-test (Performs one and two sample *t*-test on vectors of data), which is used to perform statistical analysis of differences. The significance level was set at *p* = 0.05 (*), 0.01 (**) and 0.001 (***) in the whole context. In the GWAS analysis, the *p*-value was calculated with GAMMA. Reference [69] is cited in the supplementary materials.

## Data Availability

The exome sequence data has been submitted to NCBI under the project number PRJNA550304 and is available upon the publication of the manuscript [5].

## Supplementary Materials

The following supporting information can be downloaded at: https://www.mdpi.com/article/10.3390/ijms23105587/s1**.**
