# Supplementary material for: InDels Identification and Association Analysis with Spike and Awn Length in Chinese Wheat Mini-Core Collection
Source: Int J Mol Sci. 2022 May 17;23(10):5587. doi: 10.3390/ijms23105587 (PMC9146729; doi:10.3390/ijms23105587)
Supplement: Supplementary file 1 [file ijms-23-05587-s001.zip › 01-MS_IJMS_88_supplemental figures.pdf]

International Journal of Molecular Sciences

MDPI

Supplemental figures

InDels identification and association analysis with spike and awn length in Chinese wheat mini-core collection

Zhenyu Wang [1,†], Zhongyin Deng[1,†], Xingchen Kong[1], Fang Wang[1], Jiantao Guan[1], Dada Cui[1], Guoliang Sun[1], Ruyi Liao[1], Mingxue Fu[1], Yuqing Che[1], Chenyang Hao[1], Shuaifeng Geng[1], Xueyong Zhang[1], Peng Zhou[1], Long Mao[1], Shaoshuai Liu[1,*], Aili Li[1,*]

[1] National Key Facility for Crop Gene Resources and Genetic Improvement, Institute of Crop Science, Chinese Academy of Agricultural Sciences, Beijing 100081, China

* Correspondence: liushaoshuai@caas.cn; liaili@caas.cn;

† These authors contribute equally to this work.

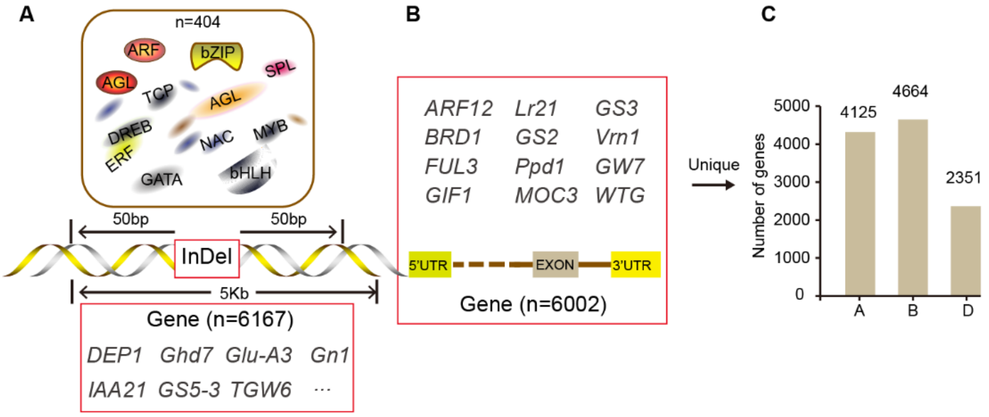

Figure S1. Distribution of the number of genes affected by potential functional InDels. (A) Cis-regulatory sequences affected by InDels, and corresponding genes. (B) Genes were affected by InDels at important positions (UTR, exon, splicing). (C) Subgenome number distribution of genes.

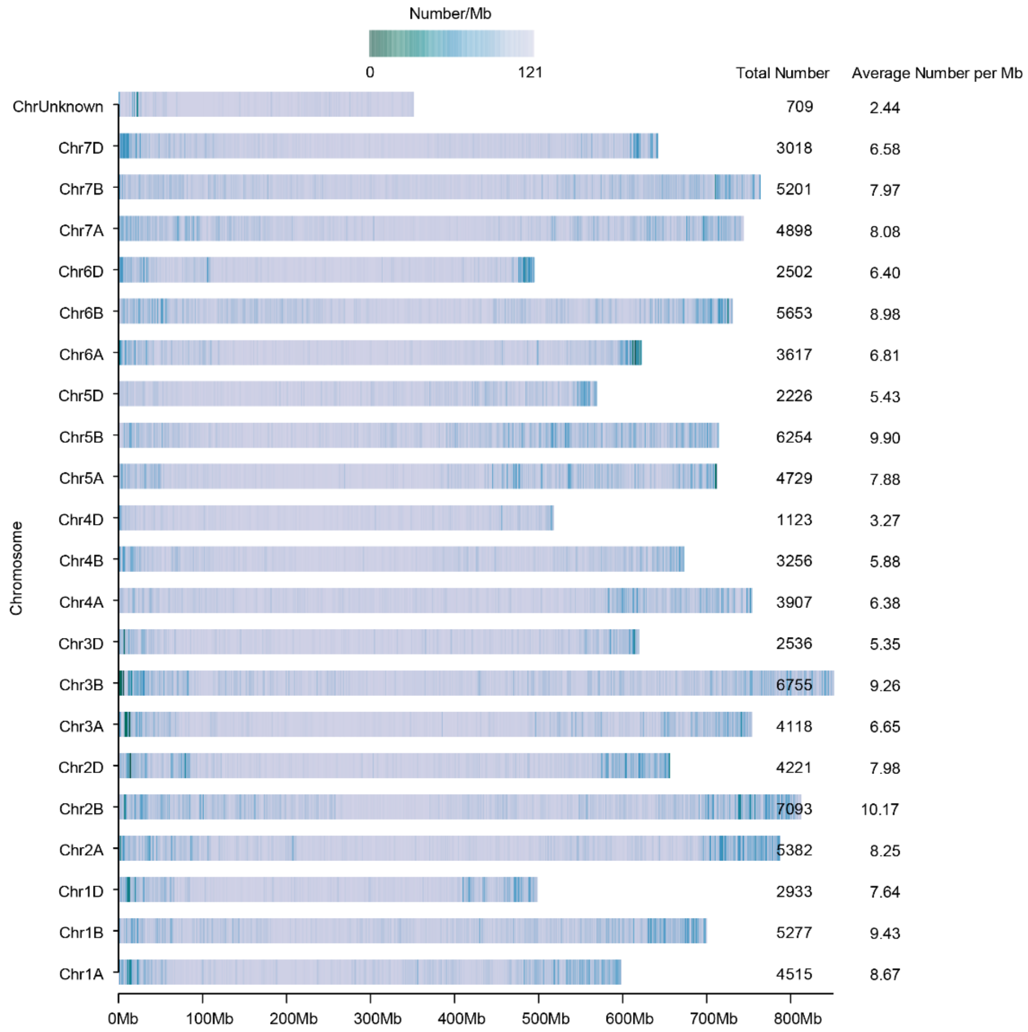

Figure S2 The density distribution of InDels along wheat chromosomes.

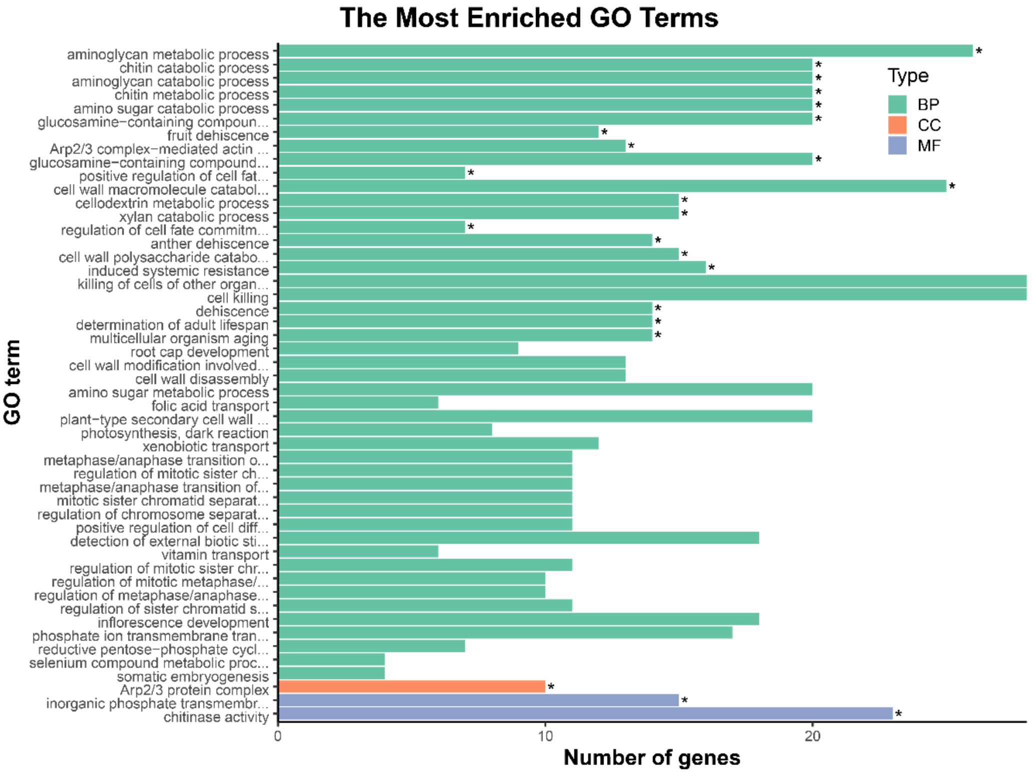

Figure S3. Bar graph of Gene Ontology (GO) enrichment analysis.

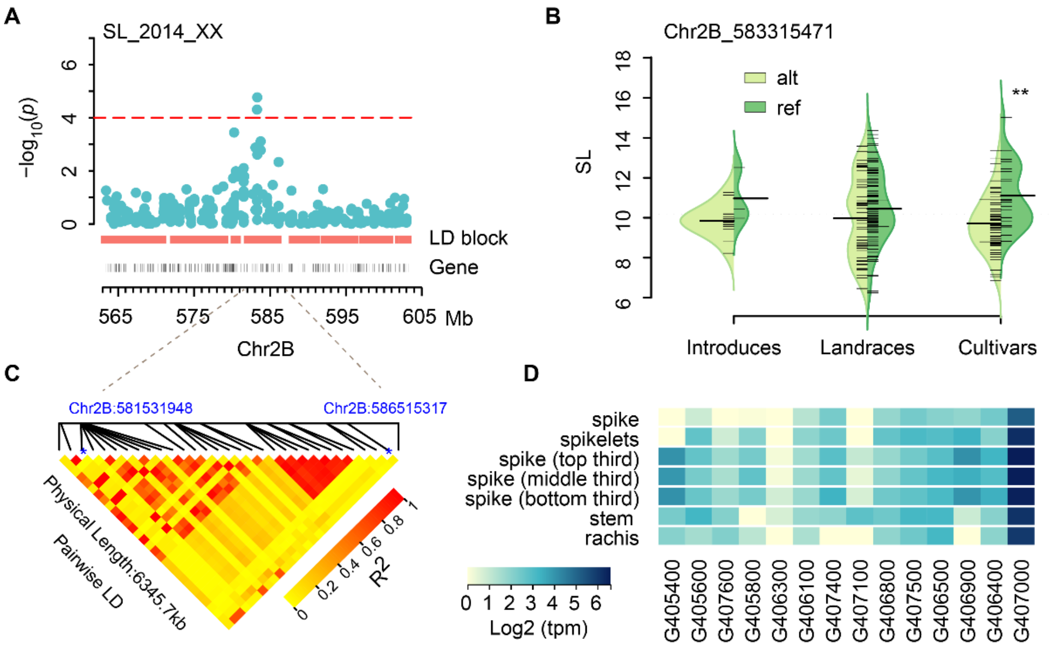

Figure S4. Identification of a major locus for spike length on chromosome 2B. (A) Manhattan plot showing the significant peak for spike length on chromosome 2B. Dashed line indicates the significance threshold ($-\log_{10}P = 4$). (B) Plant height bean plots of accessions classified by the peak InDel allele. $^{**}P < 0.01$. (C) The LD heatmap within 6.3 Mb LD block containing peak InDel. LD block border are marked using asterisk. (D) Expression heatmap of genes on the LD block.

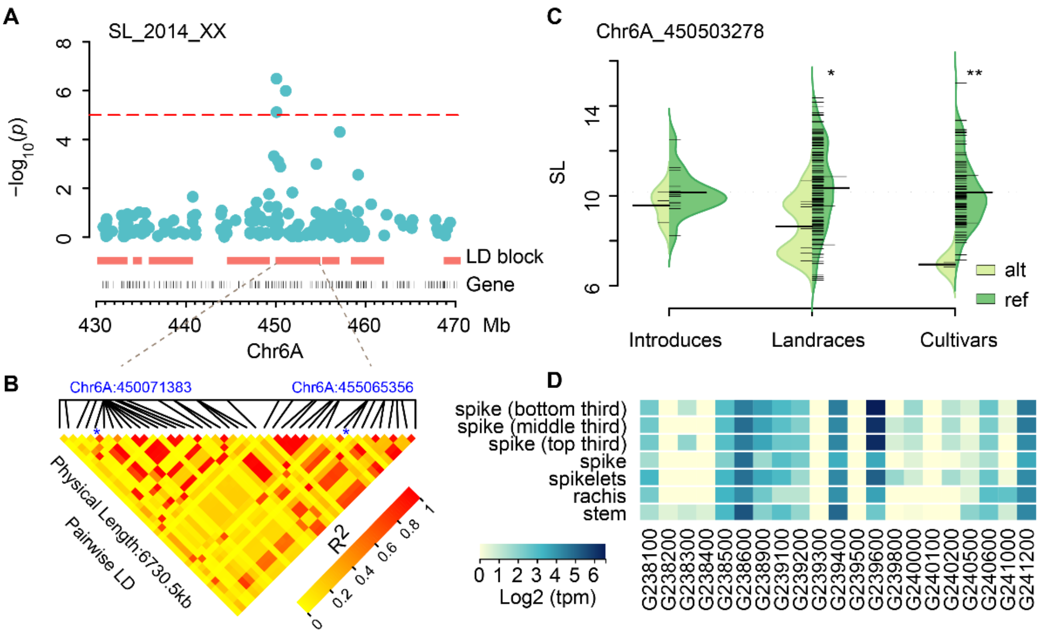

Figure S5. Identification of a major locus for spike length on chromosome 6A. (A) Manhattan plot showing the significant peak for spike length on chromosome 2B. Dashed line indicates the significance threshold ($-\log_{10}P = 4$). (B) The LD heatmap within 6.3 Mb LD block containing peak InDel. LD block border are marked using asterisk. (C) Plant height bean plots of accessions classified by the peak InDel allele. $**P < 0.01$. (D) Expression heatmap of genes on the LD block.
